# Molecular typing of *Cryptosporidium* in Israel

**DOI:** 10.1371/journal.pone.0219977

**Published:** 2019-09-03

**Authors:** Tamar Grossman, Shifra Ken-Dror, Elsa Pavlotzky, Julia Vainer, Yael Glazer, Orli Sagi, Avi Peretz, Vered Agmon, Esther Marva, Lea Valinsky

**Affiliations:** 1 Public Health Central Laboratories, Jerusalem, Israel; 2 Clalit Health Services, Haifa and Western Galilee district, Israel; 3 Division of Epidemiology, Ministry of Health, Jerusalem, Israel; 4 Soroka University Medical Center, Beer-Sheva, Israel; 5 Baruch Padeh Medical Center, Safed, Israel; 6 Bar-Ilan University, Ramat Gan, Israel; Universidade Nova de Lisboa Instituto de Higiene e Medicina Tropical, PORTUGAL

## Abstract

*Cryptosporidium* is a protozoan parasite associated with gastrointestinal illness. In immune-compromised individuals, the infection may become life-threatening. Cryptosporidiosis is a mandatory-reported disease but little was known about its prevalence and associated morbidity in Israel. Currently, laboratory diagnosis is based on microscopy or copro-antigen tests and the disease is underreported. Molecular assays, which are more sensitive and specific, are now increasingly used for identification and screening. Here, the molecular epidemiology of cryptosporidiosis is explored for the first time. Samples from 33 patients infected during an outbreak of 146 laboratory confirmed cases that occurred in Haifa and Western Galilee in 2015 were genotyped, as well as samples from 36 patients sporadically infected during 2014–2018 in different regions. The results suggest that *Cryptosporidium* subtypes found in Israel are more similar to those reported in the neighboring countries Jordan and Egypt than in European countries. *C*. *hominis* was the predominant species in the center and the north of Israel, implicating human-to-human transmission. *C*. *hominis* IeA11G3T3 was the most prevalent subtype contributing to morbidity.

## Introduction

*Cryptosporidium* infection commonly causes self-limiting 2–3 week diarrhea. In the immune-compromised or malnourished, the infection may develop into a prolonged life-threatening disease [1–4]. Young children are also more frequently and severely affected. In sub-Saharan Africa and Southeast Asia, *Cryptosporidium* is the second cause (after rotavirus) of diarrheal disease and death in children under five.[4–7]. Several factors contribute to its ability to cause outbreaks: low infection dose, faecal-oral transmission, environmentally resistant oocysts, and auto-infective cycle that leads to a high parasite load in the host and many infective oocysts shed in stool that are not host specific [6, 8].

Cryptosporidiosis was added to the list of notifiable diseases in Israel in 2001. It was then decided to collect data regarding water-transmitted pathogens for the purpose of evaluating the need to filter water entering the national drinking water network. Practitioners are required to notify all new cases to the local health districts. The 15 regional health districts and the Army Health Branch report new cases to the Division of Epidemiology of the Ministry of Health on a daily basis. Each report includes age, gender, nationality, address, and the date of disease onset. Only laboratory confirmed cases are reported. Hospital and health-maintenance organization (HMO) laboratories perform routine testing of patient samples. They are also required to notify all new cases to the local health district. In addition, they are required to send all *Cryptosporidium* positive stool samples for confirmation and molecular genotyping to the Parasitology Reference Laboratory of the Ministry of Health. If an unexpected rise in the number of cases is identified in a timely manner, the local health district may further conduct an epidemiological outbreak investigation. In the case of the outbreak described below, such investigation was not conducted.

**Fig 1:**
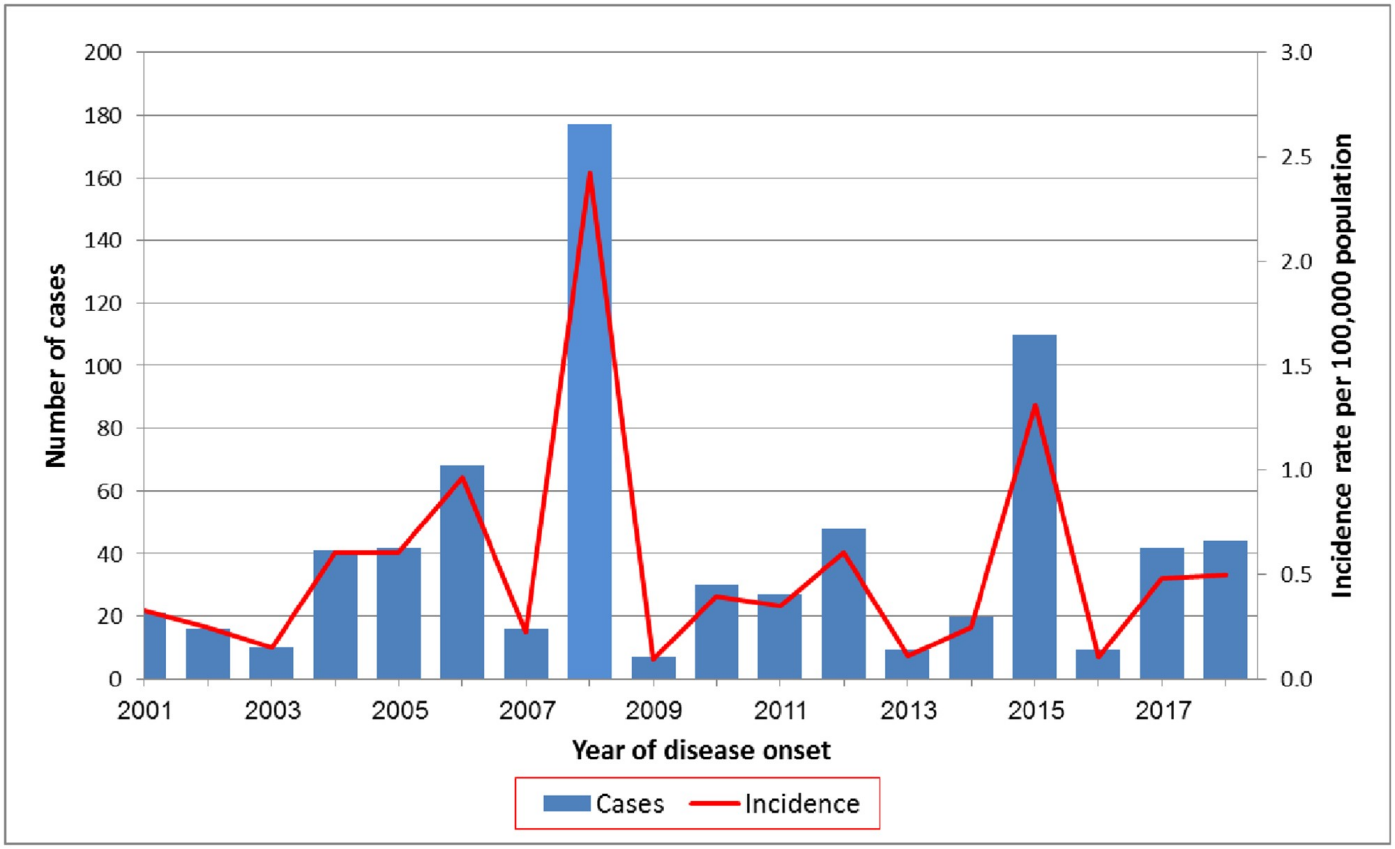
Reported *Cryptosporidium* cases in Israel and incidence rate 2001–2018.

The Division of Epidemiology reported cryptosporidiosis rates varying from 0.1 to 1 per 100,000 (7 and 68 reported cases, respectively) for most years in the period 2001–2018, with two exceptions: 177 reported cases, corresponding to infection rate of 2.4 per 100,000, in 2008, and 110 cases, 1.3 per 100,000 in 2015 (Fig 1). At both peak incidence years, 2008 and 2015, most of the reported cases originated in Haifa / West-Galilee district (157 and 82 cases, respectively). The total number of cases reported between 2001 and 2018 was 737, of whom 589 (80%) were children under five. The manual reporting system described above, although mandatory, left out of record some of the identified cryptosporidiosis cases. Notably, only 82 of the 146 outbreak cases in 2015 described in this work were included in the national database. Moreover, the reported numbers underestimated cryptosporidiosis morbidity also because of other reasons, including a frequent failure of physicians to request specific tests and the challenge of laboratory detection (see below).

The prevalence of cryptosporidiosi*s* in Israel was investigated in the 1990’s. An epidemiological study conducted in the south of Israel over one year identified *Cryptosporidium* in the stools of 3.4% of children with diarrhea and 0.7% in a control group without diarrhea living in the same area. A peak overall detection rate of 8.3% was observed in the summer months [9]. Several studies in Bedouin infants, which live in nomadic or partly nomadic communities found *Cryptosporidium* in 3–4% of stool samples. In this population, the risk of infection by the age of two was close to 49%, this was observed by monitoring stools as well as sero-positivity, [10, 11]. A sero-prevalence survey in the north of Israel demonstrated the presence of *Cryptosporidium*-specific antibodies in half of the children under the age of 12 [12]. An outbreak involving zoonotic transmission was also identified [13]. Additional studies revealed morbidity caused by *Cryptosporidium* in hospitalized children. Cryptosporidiosis was found in children with diarrhea after solid organ transplantation at the Schneider Children’s Medical Center [14]. A recent study of children hospitalized at the Padeh-Poriya Medical Center, located in the north of Israel, found *Cryptosporidium* to be the second most common cause of gastrointestinal disease after *Campylobacter* [15]. Although the total number of positive specimens in this study was small, two *Cryptosporidium* species, *Cryptosporidium hominis* (*C*. *hominis*) and *Cryptosporidium parvum* (*C*. *parvum*), were identified [15].

Laboratory diagnosis of cryptosporidiosi*s* is based primarily on stool samples. Currently, most clinical laboratories perform microscopic examination of ova and parasites. Microscopic examination, lacks sensitivity because *Cryptosporidium* oocysts are transparent and easily missed [16, 17]. Most clinical laboratories in Israel perform additional diagnostic tests only when these are specifically requested by the physician. These tests include mainly copro-antigen tests and/or modified Ziehl–Neelsen stain. In recent years, the application of molecular techniques with high sensitivity and specificity is gradually increasing. Quantitative real-time PCR (qPCR) for detection of *Cryptosporidium* from stool samples is now used in the Parasitology Reference Laboratory of the Ministry of Health and is currently in stages of evaluation and implementation in other primary laboratories in Israel. The feasibility of using a molecular diagnostic approach was evaluated in a study performed in Haifa and West Galilee during November 2013 –April 2014 [18]. The performance of a commercial gastrointestinal panel (NanoCHIP1, Savyon Diagnostics, Ashdod, IL) was compared to that of conventional methods. This study found 100% agreement in identification of *Cryptosporidium* in faecal samples from symptomatic patients. It should be noted, though, that during that period only two samples out of 161 tested were positive.

*Cryptosporidium* has a worldwide distribution. Most infections are caused by two species, *C*. *hominis*, for which humans are largely the main host, and *C*. *parvum*, which is zoonotic and has a plethora of suitable host species. Together, these species account for 90% of human infection [19, 20]. The largest outbreaks of cryptosporidiosis in humans, involving tens to hundreds of thousands of people, have been caused by contamination of drinking water [8]. Species determination and subtyping has contributed to understanding of the epidemiology of cryptosporidiosi*s* in outbreaks as well as in sporadic infections [6, 19–21].

Although reporting of cryptosporidiosis is mandatory in Israel, little is known about the prevalence and associated morbidity of the disease. An outbreak of 146 cases of cryptosporidiosi*s* occurred in Haifa and West-Galilee in the summer of 2015 and the molecular typing of samples from 33 patients, along with 36 samples from sporadic infections, enabled us to explore the molecular epidemiology of cryptosporidiosi*s* in Israel for the first time.

## Materials and methods

**Fig 2:**
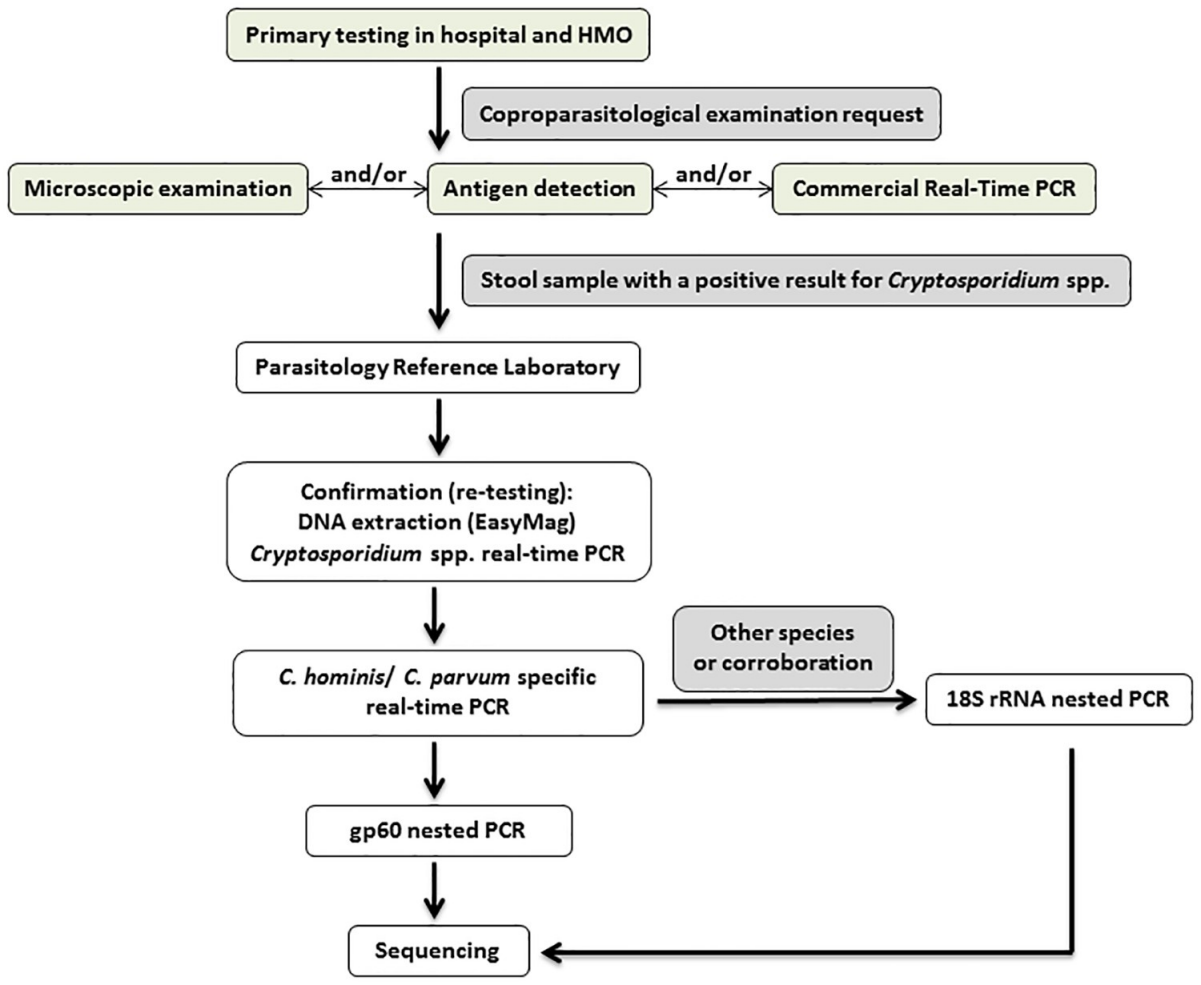
Diagnosis and molecular genotyping.

A flow diagram describing the algorithm for diagnosis and molecular genotyping of cryptosporidiosis is described in Fig 2.

Primary testing in hospital and HMO laboratories: Stool samples from patients with diarrhea were subjected to routine testing in hospital and HMO laboratories. All stools were subjected to microscopic examination of ova and parasites, consisting of direct examination of wet mounts as well as concentration method. Complementary tests specific for *Cryptosporidium* were conducted only upon specific requests from the physicians, or when microscopic examination in the laboratory raised suspicion. In most facilities, those tests included antigen detection (*Cryptosporidium*-Strip, Coris BioConcepts, Gembloux, Belgium) and/or modified acid fast staining. A new commercial multiplex qPCR gastro-intestinal parasite panel (Allplex, Seegene), including *Cryptosporidium*, has been evaluated at the central laboratory of Haifa and Western Galilee HMO. During the 2015 outbreak, 7554 stools from patients with gastrointestinal symptoms were tested at Haifa and Western Galilee HMO, of them 146 (1.9%) were found positive for *Cryptosporidium*. Thirty out of those were tested and identified using the evaluated molecular assay.

Post examination at the hospital and HMO laboratories: Faecal samples positive for *Cryptosporidium* left over from testing, were kept frozen until sent to the Parasitology Reference Laboratory for re-testing and genotyping.

Confirmation and genotyping in the Parasitology Reference Laboratory: DNA was extracted from 100 mg stool via NUCLISENS easyMAG platform (bioMérieux), according to the protocol published by Jeddi et al. [22]. Elution volume was 110 μl. qPCR tests were implemented for detection and genotyping of *Cryptosporidium* spp. One qPCR assay amplifies a region of the 18S rRNA sequence common to most *Cryptosporidium* spp. and is used for the detection of *Cryptosporidium* spp. [23]. This assay was previously validated by the developers [23] as well as by others [24]. All samples (36 from the outbreak and 40 sporadic cases) were subjected to this assay.

qPCR reactions were prepared in a final volume of 50 μl containing 5 μl of genomic DNA, 0.5 μM of the primer set JVAF and JVAR, 0.25 μM of the probe JVAP18S (S1 Table) and 25 μl ABsolute Blue qPCR Low ROX Mix (ThermoFisher Scientific, Lithuania). The amplification protocol included an initial hold step of 15 min at 95 °C followed by 52 cycles of 15 s at 95 °C and 1 min at 60 °C and was carried out on a 750 Applied Biosystem qPCR system (Rhenium). Appropriate positive, negative, and inhibition controls were routinely included in each round of qPCR assays.

Another qPCR assay that is based on a different region of the 18S rRNA sequence and can specifically target *C*. *hominis* and *C*. *parvum* was used for genotyping [25]. This assay cannot differentiate *C*. *hominis* from the closely related but rare species *C*. *cuniculus*; differentiation necessitates sequencing of a region of the 18S rRNA gene or the 60kDa glycoprotein (*gp60*) gene [26, 27] (see below). All samples (36 from the outbreak and 40 sporadic cases) were subjected to this assay. The qPCR reactions were conducted in a monoplex format, each prepared in a final volume of 20 μl containing 2 μl of genomic DNA, 0.5 μM of the primer set Mary-F and Mary-R, 0.25 μM of one of the probes Pan-Crypto probe, *C*. *hominis* probe or *C*. *parvum* probe (S1 Table) and 25 μl ABsolute Blue qPCR Low ROX Mix (ThermoFisher Scientific, Lithuania). The amplification protocol was the same as for the *Cryptosporidium* spp. qPCR assay described above. Appropriate positive, negative, and inhibition controls were routinely included in each round of qPCR assays.

The qPCR test results were corroborated on two sequences from patients in the sporadic group by performing nested PCR and sequencing a 0.6 kb segment of the 18S rRNA gene according to Silva et al. [28]. PCR reactions were conducted with PCR-Ready High Specificity mix (Syntezza Bioscience Ltd., Israel) in a final volume of 25 μl consisting of 2μl of genomic DNA and 0.5μM of the primers pairs SHP1/SHP2 in the primary reaction and SHP3/SSU-R3 in the secondary reaction (S1 Table). Cycling parameters for the primary PCR reaction were an initial step of 94 °C for 3 min, followed by 39 cycles of 94 °C for 45 s, 56 °C for 45 s, and 72 °C for 70 s with a final extension of 72 °C for 7 min. The same conditions were used in the secondary PCR.

Identified C. *hominis* or *C*. *parvum* single-infection samples were further subtyped by amplification and sequencing of both strands of an amplicon of about 0.9 kb of the *gp60* gene, according to a nested PCR protocol by Feng et al. [29]. PCR reactions were conducted with PCR-Ready High Specificity mix (Syntezza Bioscience Ltd., Israel) in a final volume of 50 μl consisting of 5 μl of genomic DNA and 0.5 μM of the primers pairs LX0374/ LX0375 in the primary reaction and AL3534/AL3532 in the secondary reaction (S1 Table). Cycling parameters for the primary PCR reaction were an initial step of 94 °C for 3 min, followed by 39 cycles of 94 °C for 45 s, 52 °C for 45 s, and 72 °C for 70 s with a final extension of 72 °C for 7 min. The same conditions were used in the secondary PCR. In a few cases where amplification failed, a smaller segment was amplified and sequenced according to Sulaiman et al. [30]. PCR reactions were conducted with PCR-Ready High Specificity mix (Syntezza Bioscience Ltd., Israel) in a final volume of 40 μl consisting of 2 μl of genomic DNA and 0.5 μM of the primers pairs AL3531/AL3533 in the primary reaction and LX0029/ AL3532 in the secondary reaction (S1 Table). The AL3532 primer is common to both *gp60* assays. Cycling parameters for the primary PCR reaction were an initial step of 94 °C for 3 min, followed by 39 cycles of 94 °C for 45 s, 46 °C for 45 s, and 72 °C for 45 s with a final extension of 72 °C for 7 min. The same conditions were used in the secondary PCR. Amplification of the *gp60* gene was successful in stool samples from 33 outbreak patients and 36 sporadic.

PCR products were sequenced using internal primer sets. Sequencing was performed at the Center for Genomic Technologies, Institute of Life Sciences, the Hebrew University of Jerusalem, using BigDye Terminator v1.1 chemistry (Applied Biosystems, Foster City, California, USA). Sequence assemblage to create consensus sequences and alignments, were performed using the BioNumerics v 7.6 software (Applied Maths, Kortijk, Belgium). The BLAST tool (http://blast.ncbi.nlm.nih.gov/Blast.cgi) was used to compare nucleotide sequences with sequences deposited in the NCBI. Patient residence plotting was performed using Microreact [31].

All these procedures were part of routine health care and of reference lab duty code regulated (in general terms) by law (Public Health Ordinance, 1940). The study was approved by the Clalit Health Services Ethics Committee, which stipulated that the study did not need informed consent.

## Results

**Fig 3:**
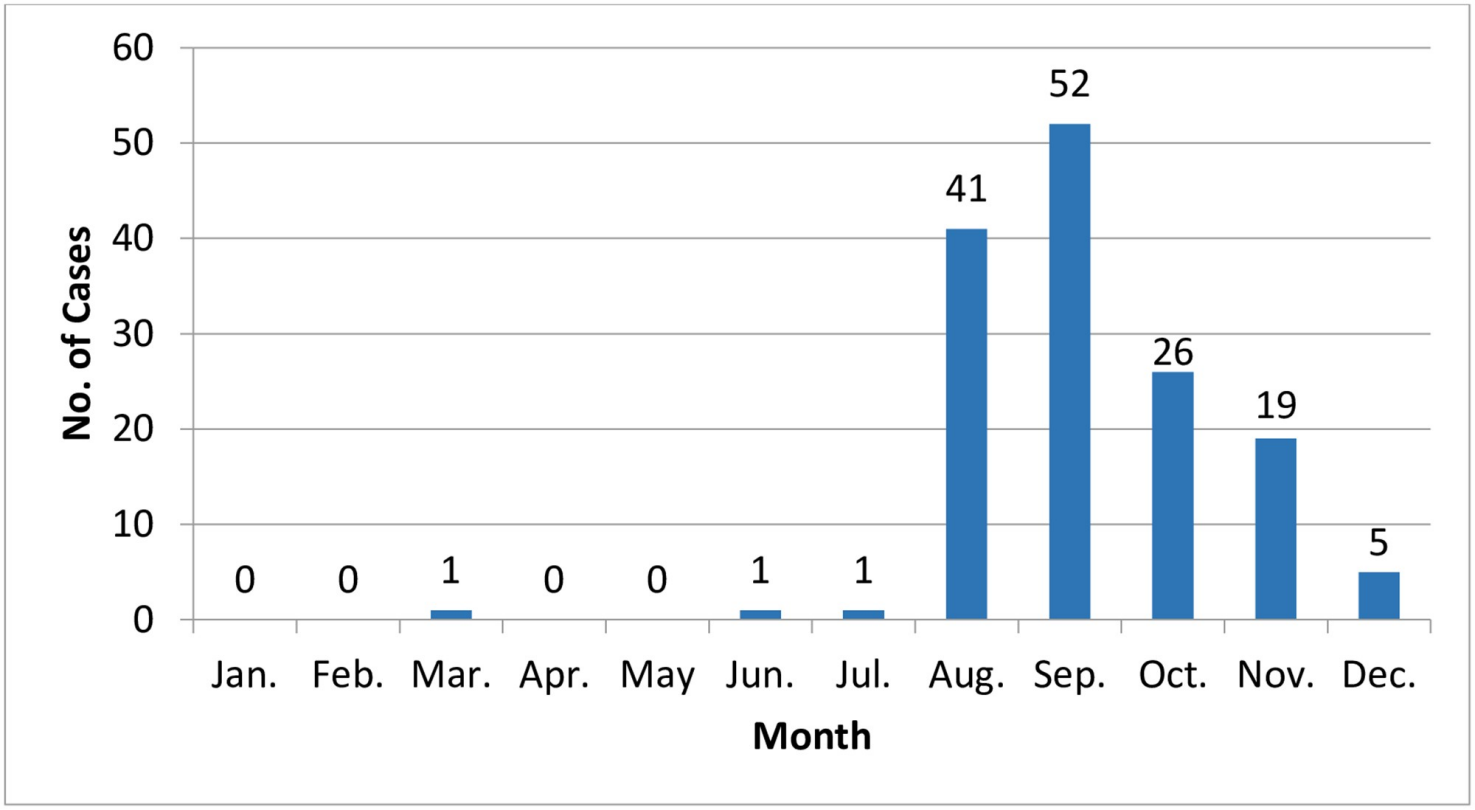
*Cryptosporidium* cases in Haifa and West Galilee per month during 2015.

**Fig 4:**
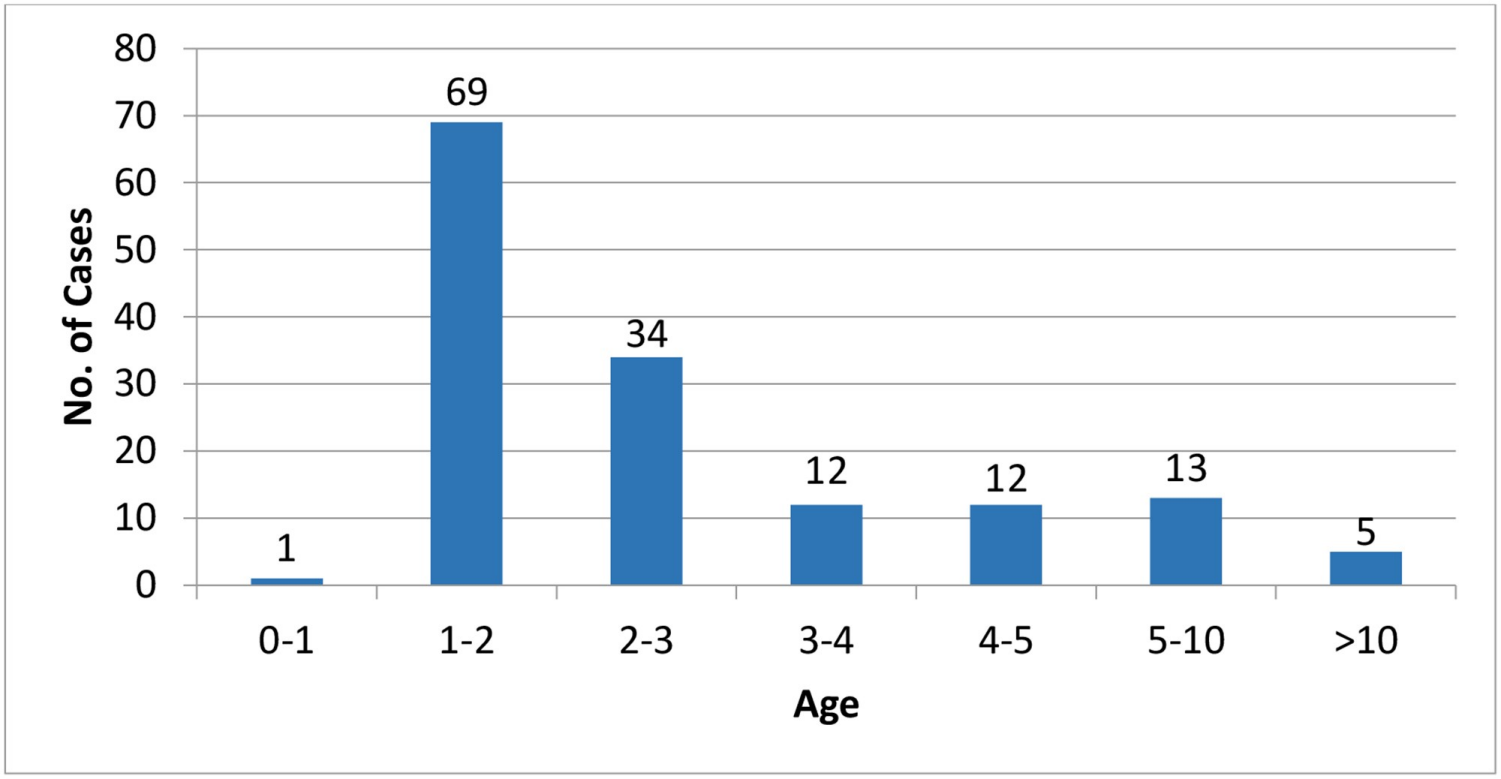
Age distribution of *Cryptosporidium* cases in Haifa and West Galilee during 2015.

Between August and December 2015, the Haifa and West Galilee HMO identified an increase in *Cryptosporidium* infection rates (see Discussion), constituting an outbreak of 146 laboratory confirmed cases in the district (Fig 3). A majority were young children: 70% (104) were under the age of three and 88% (128) under five (Fig 4); 59% (86) were male. Stool samples from these patients were subjected to parasitological examination. They were identified positive for *Cryptosporidium* using microscopic and antigen detection (116 patients), or molecular tests (30 patients). In addition to *Cryptosporidium*, 21 (14%) had other parasitological infections: 10 had *Dientamoeba fragilis* (*D*. *fragilis*), 3 *Blastocystis* sp., 2 a mixed infection of *D*. *fragilis* and *Blastocystis* sp. and 6 had *Giardia duodenalis*.

**Fig 5:**
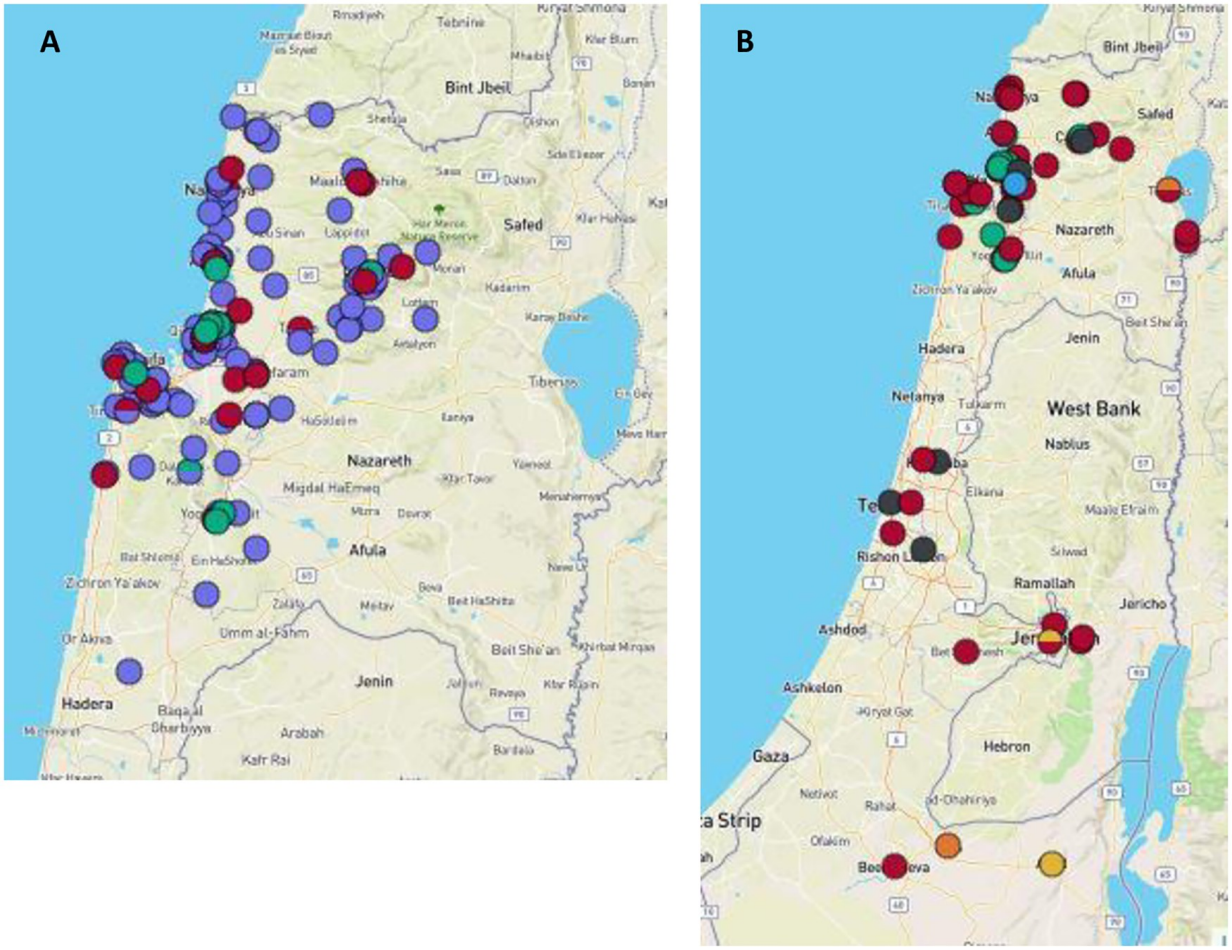
The geographic distribution and genotypes of *Cryptosporidium* cases in Israel. A. Residence of the 2015 outbreak patients (N = 146), ploted on Haifa and West Galilee geographic map. B. Residence of genotyped patients. Purple, identyfied at the HMO but not genotyped; Red, *C*. *hominis* **IeA11G3T3**; Green, *C*. *hominis* **IbA6G3**. Grey, *C*. *hominis* **IdA16;** Light Blue, *C*. *hominis* **IbA10G2**; Yellow, *C*. *parvum* **IIdA20G**; Orange, *C*. *parvum* **IIaA15G2R1**. Maps were generated using Microreact [31]; A dynamic version that includes the tested determinants can be found at Microreact https://microreact.org/project/1hgtRJKlg/4da49c44 and https://microreact.org/project/HD3sxQKDh/350ce02e for Fig 5A and Fig 5B, respectively.

Of the stool samples that were found positive for *Cryptosporidium* at the Haifa and West Galilee HMO lab, 36 samples from 34 patients had sufficient stool remaining and were sent to the Parasitology Reference Lab for genotyping. Those were re-tested for the presence of *Cryptosporidium*, in accordance to the reference lab work flow, and genotyped. Analysis of the 18S rRNA gene revealed that all positive samples contained *Cryptosporidium* of the species *C*. *hominis*. Further sequencing of the *gp60* gene was successful in stools from 33 patients. Two subtypes of *C*. *hominis* were identified according to the scheme developed by [30], **IeA11G3T3** in 23 patients, and **IbA6G3** in 10. The geographic distribution of patient residence and subtypes in this outbreak is shown in Fig 5A.

In addition to samples from the outbreak, 40 stool samples from 40 sporadic *Cryptosporidium*-positive patients collected in other regions throughout Israel between 2014 and 2018 were sent to the reference lab and analyzed as described above. Three were positive for *C*. *hominis* but further analysis of the *gp60* subtype was not successful. Another patient with a mixed infection of *C*. *hominis* and *C*. *parvum* was also not further subtyped. Thirty-six patients were successfully subtyped. The *Cryptosporidium* subtype in 24 patients was *C*. *hominis* **IeA11G3T3,** 7 had *C*. *hominis* **IdA16** and one was *C*. *hominis* **IbA10G2**. Four patients had *C*. *parvum*, two were subtype **IIdA20G** and two **IIaA15G2R1**. The geographic distribution of all cases with identified subtype is shown in Fig 5B. *Cryptosporidium* species and subtypes found in this study are summarized in Table 1. All sequencing results were deposited in the GenBank. Sequences of the *gp60* gene from the outbreak and sporadic cases have accession numbers MK095273-MK095305 and MK095306-MK095341, respectively. The 18S rRNA sequences have accession numbers MK801120 and MK801121.

**Table 1:** *Cryptosporidium* subtypes.

Species	Subtype	2015 Outbreak cases	Sporadic cases	Comments	General distribution
*C*. *hominis*	**IeA11G3T3**	23	24	dominant	sporadic worldwide
*C*. *hominis*	**IbA6G3**	10	─	outbreak only	Egypt, Jordan, Australia; rarely reported
*C*. *hominis*	**IbA10G2**	─	1		prevalent worldwide, dominant in Europe
*C*. *hominis*	**IdA16**	─	7		Australia, India and China, sporadic
*C*. *parvum*	**IIaA15G2R1**	─	2		prevalent worldwide
*C*. *parvum*	**IIdA20G**	─	2		prevalent in Egypt

## Discussion

Early studies conducted until 2001 identified cryptosporidiosis as a significant cause of diarrhea in children in the south and north of Israel, especially in summer [9, 10, 12]. *Cryptosporidium* also caused morbidity after solid organ transplantation in a pediatric hospital [14]. A recent study reported *Cryptosporidium* to be the second most common cause of gastrointestinal disease after *Campylobacter* in hospitalized children. Both *C*. *hominis* and *C*. *parvum* were found in those patients [15]. Presence of *C*. *hominis*, *C*. *parvum*, *C*. *andersoni* and C. *muris* in wastewater effluents was reported previously [32]. The present study provides new information about prevalence, morbidity and species and subtype distribution.

*Cryptosporidium* infections are generally under-detected and underreported, especially in developing countries [8]. In Israel, most clinical laboratories of the HMOs and hospitals perform copro-antigen tests and microscopic detection tests for *Cryptosporidium* upon physician’s request or suspicion following initial examination of the sample, but these methods are not sensitive, often resulting in false negative identification. Molecular assays, highly sensitive and specific [17, 33], are now being evaluated and introduced into clinical laboratories Thus, a corresponding increase in detection rates is expected as well as improved surveillance.

*Cryptosporidium* causes outbreaks of gastrointestinal illness worldwide [5, 7, 21, 34, 35]. An outbreak of 146 laboratory-confirmed cases was identified between August and December 2015 in the Haifa and West Galilee district of Israel (Fig 3). Of those, 104 were 3 years old or younger, and a total of 127 (88%) were 5 or younger (Fig 4). Morbidity in children is typical for cryptosporidiosis since the pediatric group is generally more susceptible to infection and illness [2]. Adults tend to be less symptomatic and therefore do not often seek medical assistance and are less likely to be diagnosed. National data shows a previous peak of cryptosporidiosis in 2008 (Fig 1). As in 2015, most of those cases originated in Haifa and West Galilee district, together predicting recurrences in the future.

The main transmission pathways of *C*. *hominis* are food and water contamination as well as person to person. Outbreaks that originate from contaminated drinking water have the potential of causing morbidity in thousands of people. Until 2016 drinking water in Israel came from a main water body (Lake of Galilee), supplemented with water from small surface water springs and deep ground water. In 2007 drinking water begun to be centrally filtered. In 2016 a new system of desalination of Mediterranean sea water via reverse-osmosis was introduced and presently, most drinking water originates from this system. Regulations regarding raw water produced in surface water facilities mandate at least two orders of magnitude reduction in *Cryptosporidium* oocyst concentration (99% removal) by filtration. If high concentrations are suspected to exist, health authorities may request removal of more than 99% (public health regulations 2013). Drinking water originating in Lake Galilee, springs and ground water connected to springs are monitored for the presence of *Cryptosporidium* oocysts and other pathogens. Regulations regarding lake Galilee demand bi-monthly testing of both raw water and treated-water. Post-treatment *Cryptosporidium* concentrations of less than 1 oocyst/10 liters are considered adequate. Higher levels must be reported to the Ministry of Health, in parallel to repeating the monitoring continuously. Preventive actions are taken as necessary (Ministry of Health, regulations for testing and monitoring of drinking water, 2018). In accordance with the United States Environmental Protection Agency (US EPA) method 1623.1, detection of *Cryptosporidium* oocysts in water consists of several sequential steps: filtration, immunomagnetic separation, and identification using fluorescence-conjugated antibodies and microscopy. Identification is completed with Dapi staining and Differential Interference Contrast (DIC) microscopy.

Between 2007 and now, low oocyst levels were consistently demonstrated, mostly with average concentration of less than 1 oocyst/10 liters (Israel National Water Surveillance Agency, personal communication), which is also in accordance with British standard [36]. In particular, sampling of drinking water supplied to the Haifa and West Galelee area between January and December 2015 did not demonstrate abnormal levels. None of the methods used to remove oocysts from public drinking water is however completely effective, besides, only a subset of drinking water are tested each year. Therefore, it is not possible to rule out a small, undetected breach in a filter.

A transmission pathway known to cause outbreaks in other countries, which also contributes to a seasonal increase in sporadic cryptosporidiosis during the summer months is the use of recreational waters [37–39]. In outbreaks in England and Wales that occurred between 2009 and 2017, recreational waters were the leading cause, involved in 46% of the outbreaks [37]. The species *C*. *hominis* was found responsible for 88% out of those. The second cause was animal contact (42%), with the major species identified being *C*. *parvum*, responsible for 77% of those [37].

**Fig 6:**
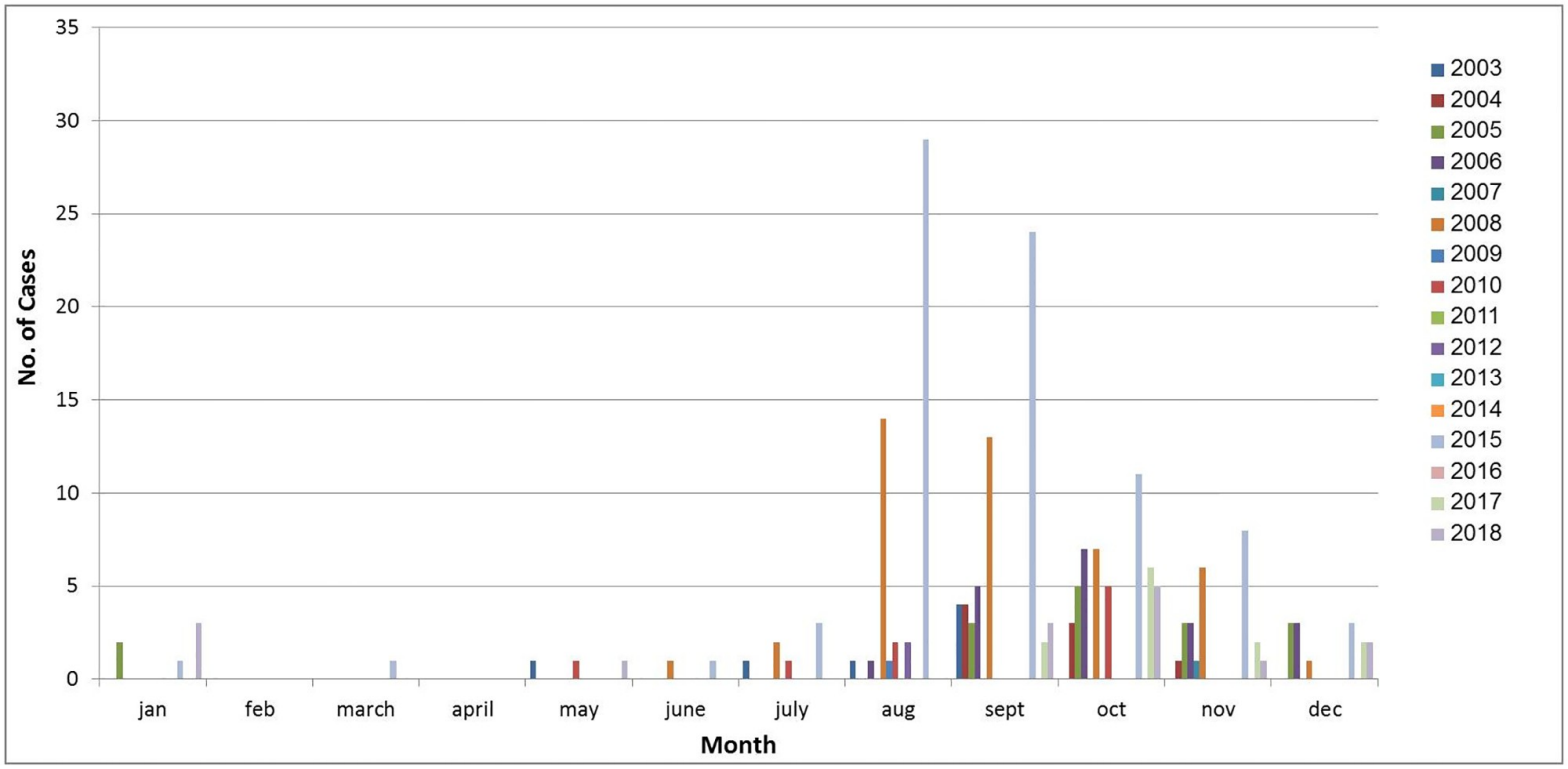
*Cryptosporidium* cases per month in Haifa and West Galilee HMO 2003–2018. The number of cases per month in the years 2003–2018 are shown (national database).

Unexpected peaks of *Cryptosporidium* cases have been described in several European countries. A simultaneous 2 to 5 fold increase of *Cryptosporidium* infections, compared to previous years, occurred in the Netherlands, the United Kingdom and Germany in the late summer season of 2012 [40]. In the UK, travel abroad was identified as a risk factor; in the Netherlands, there was an association to bottled water. However, no single source could fully explain the increase of cryptosporidiosis. A combination of multiple factors, like weather conditions and person-to-person transmission, may have contributed [40]. A seasonal autumn peak trend had been previously noted in the UK, with *C*. *hominis* found predominantly in individuals who used swimming pools [41]. In Spain a seasonal autochthonous summer peak and a smaller peak in autumn were identified in 2015, a trend that was not observed in the following year. In this case, as well, a common source was not identified [42]. Analysis of reported cases per month from the Haifa -West Galilee district during the years 2003–2018 showed a seasonal increase, in August through December (Fig 6). The outbreak in 2015 coincides with the seasonal pattern, but the number of cases was about 9 times higher than expected. With exception of 2008 and 2015, the total number of cases from Haifa and West Galilee HMO between 2001 and 2018 as reported to the national database was on average 9 cases per year, while in 2015, 82 cases were reported from the same area. With all this in mind, the 2015 outbreak could have involved transmission in swimming pools, which are very popular in summer and/or person to person contamination in kindergartens.

A limitation of this study is that an epidemiological investigation including the testing of environmental and animal samples was not conducted. Consequently, the reason for the increment in cases remains unknown.

Samples from 33 (23%) patients out of the 146 diagnosed during the outbreak were available and successfully genotyped. All were found to be infected with *C*. *hominis*. Of these, 23 had subtype **IeA11G3T3**, and 10 had **IbA6G3.** *C*. *hominis* subtype **IbA6G3** was found only in patients from this outbreak.

In addition to the outbreak cases, 36 of 40 stools of sporadic cases from different parts of Israel were successfully subtyped. In 24 *C*. *hominis* **IeA11G3T3** was identified, indicating probable dominance of this type in Israel. Within the **Ie**-subtype family, **IeA11G3T3** is the predominant subtype worldwide [43]. This subtype was detected in sporadic cryptosporidiosis, e.g. in Sao Tome and Principe (West Africa), Australia, India, Kuwait, Peru, Mexico and Spain but is not the major subtype in these countries [30, 44–48]. If *C*. *hominis* **IeA11G3T3** continues to be prevalent in Israel, multi-locus genotyping may be required for effective epidemiological surveillance [4, 49].

Subtype *C*. *hominis* **IbA10G2** is highly prevalent worldwide, and in Europe it is predominant as a cause of cryptosporidiosi*s* in humans [4, 49, 50]. This subtype has been identified in Israel only in one recent sample (in 2018) from a patient living in the West Galilee area. The **Ib**-subtype family is generally considered potentially more virulent [20, 51]; in this study, we identified another member of this family, *C*. *hominis* **IbA6G3**, in 10 outbreak patients. However, virulence assessment was not possible since information regarding the course of disease was lacking. Worldwide, *C*. *hominis* **IbA6G3** has been rarely reported. To our knowledge, it has been observed so far in human isolates only in Jordan, Egypt and Australia [48, 52, 53].

Among sporadic cases there were 7 *C*. *hominis* **IdA16**, a subtype reported to have caused sporadic cryptosporidiosis in Australia, India and China [29, 45, 54].

Two isolates from the south of Israel were *C*. *parvum*, subtype **IIdA20G**. This subtype was found to have significant prevalence in several provinces in the north of Egypt in both humans and livestock [55]. Other two isolates were *C*. *parvum*, subtype **IIaA15G2R1**, a prevalent subtype in humans as well as in dairy cattle worldwide, including Egypt [53, 56].

In summary, our study shows that *Cryptosporidium* is endemic in Israel and is responsible for outbreaks as well as sporadic morbidity. Preliminary data suggest that *C*. *hominis* is the main species in central and north Israel, implicating human-to-human transmission. The genotypes of *Cryptosporidium* resemble those found in Israel’s neighboring countries Jordan and Egypt more than those found in Europe. Several *C*. *hominis* subtypes contribute to morbidity, with **IeA11G3T3** being the most prevalent across the country. *C*. *parvum* was also detected.

This is the first report on the genetic and demographic characteristics of cryptosporidiosis in Israel. The combination of molecular-epidemiology analysis with the demographic metadata can reveal regional transmission routes and infection sources, prompting management of water systems and livestock. Implementing sensitive molecular assays into primary care are expected to increase the number of cases identified and create a larger base of molecular information. New genomic-based technologies to further investigate the molecular epidemiology of *C*. *hominis* **IeA11G3T3**, which appears to be the dominant strain in Israel, are essential.

## Supporting information

S1 Table Oligonucleotides used for the molecular identification and/or characterization. (DOCX)
