# Supplementary material for: Molecular typing of Cryptosporidium in Israel
Source: PLoS One. 2019 Sep 3;14(9):e0219977. doi: 10.1371/journal.pone.0219977 (PMC6721021; doi:10.1371/journal.pone.0219977)
Supplement: S1 Table — (DOCX) [file pone.0219977.s001.docx]

| **Assay description** | **Locus** | **Oligonucleotide** | **Sequence (5'–3')** | **Reference** |
| --- | --- | --- | --- | --- |
| *Cryptosporidium* spp. RT-PCR | 18S rRNA | JVAP18S probe | FAM-CGCGCCTGCTGCCTTCCTTAGATG-BHQ | 23 |
|  |  | JVAF | ATGACGGGTAACGGGGAAT |  |
|  |  | JVAR | CCA ATTACAAAACCAAAA AGTCC |  |
| *C. hominis/ C. parvum* specific RT-PCR | 18S rRNA | Pan-Crypto probe | FAM-CTAGAGCTAATACATGCGAAAAAA-MGB | 25 |
|  |  | *C. hominis* probe | FAM-ATCACAATTAATGT-MGB |  |
|  |  | *C. parvum* probe | VIC-ATCACATTAAATGT-MGB |  |
|  |  | Mary-F | CATGGATAACCGTGGTAAT |  |
|  |  | Mary-R | TACCCTACCGTCTAAAGCTG |  |
| 18S rRNA nested PCR | 18S rRNA | SHP1 | ACCTATCAGCTTTAGACGGTAGGGTAT | 28 |
|  |  | SHP2 | TTCTCATAAGGTGCTGAAGGAGTA AGG |  |
|  |  | SHP3 | ACAGGGAGGTAGTGCAAGAAATAACA |  |
|  |  | SSU-R3 | AAGGAGTAGGAACAACCTCCA |  |
| *gp60* nested *PCR* | *gp60* | LX0374 | TTACTCTCCGTTATAGTCTCC | 29 |
|  |  | LX0375 | GGAAGGAACGATGTATCTGA |  |
|  |  | AL3534 | GCAGAGGAACCAGCATC |  |
|  |  | AL3532 | TCCGCTGTATTCTCAGCC |  |
| *gp60 nested PCR* (short) | *gp60* | AL3531 | ATAGTCTCCGCTGTATTC | 30 |
|  |  | AL3533 | GAGATATATCTTGGTGCG |  |
|  |  | LX0029 | CGAACCACATTACAAATGAAGT |  |
|  |  | AL3532 | TCCGCTGTATTCTCAGCC |  |
